# The complete chloroplast genome of *Carpinus hebestroma*, a critically endangered species endemic to Taiwan

**DOI:** 10.1080/23802359.2018.1481784

**Published:** 2018-06-18

**Authors:** Gaini Wang, Ying Li

**Affiliations:** State Key Laboratory of Grassland Agro-Ecosystem College of Life Sciences, Lanzhou University, Lanzhou, China

**Keywords:** *Carpinus hebestroma*, chloroplast genome, phylogenetic analysis

## Abstract

*Carpinus hebestroma* (Betulaceae) is a critically endangered species endemic to Taiwan. In this study, we assembled the complete chloroplast (cp) genome of *C. hebestroma* and the cp genome was 159,231 bp in length, including a large single copy (LSC) region of 88,168 bp, a small single copy (SSC) region of 18,874 bp and a pair of inverted repeats (IRs) of 52,189 bp. The genome contained 124 genes, including 85 protein-coding genes, 30 tRNA genes and 8 rRNA genes. The overall GC content of the chloroplast genome was 36.46%. A phylogenetic analysis demonstrated a close relationship between *C. hebestroma* and *Ostrya rehderiana*.

*Carpinus hebestroma* Yamam is a species of small tree within the family Betulaceae, and this species was endemic to Taiwan and known only from the type specimen locality, in Batakan, Taiwan, China. Within this locality, the species has small and fragmented subpopulations, and the population was still declining because of the landslides after typhoons. This species was listed as critically endangered species in the IUCN red list now (http://www.iucnredlist.org/details/194577/0). Therefore, measures of conservation and restoration are urgently needed. The plastid genome will contribute to develop protection measures for this endangered species.

Fresh leaves were collected from an individual of *C. hebestroma* in Taiwan (24°09′17″ N, 121°29′33″ E; China; voucher 2015-Taiwan-001 deposited in the herbarium of Lanzhou University, Lanzhou, China). Genomic DNA was isolated using the modified CTAB method (Doyle 1987). The whole-genomic DNA data were sequenced using the Illumina Hiseq 2500 platform (Illumina, San Diego, CA). The paired-end data set comprised 72.2 million reads (2 × 150 bp). Chloroplast genome assembly using the Fast-Plast pipeline (https://github.com/mrmckain/Fast-Plast; McKain and Wilson, unpublished). Annotation was performed with Plann (Plastome Annotator, Huang and Cronk 2015).

The complete chloroplast genome of *C. hebestroma* (GenBank: MG720819) was 159,231 bp in length, comprising a LSC of 88,168 bp and a SSC of 18,874 bp, separated by IRs regions of 52,189 bp. It contained 124 genes, including 85 protein-coding genes, eight rRNA genes, and 30 tRNA genes. The plastome contained 95 unique genes, 14 genes duplicated in the IR regions. Among annotated genes, nine genes contained a single intron, and four genes had two introns (e.g. *clp*P, *ycf*3, *rps*12 and *rpl*2). The base compositions of major chloroplast genome were uneven (31.34% A, 18.60% C, 17.86% G and 32.20% T), with an overall GC content of 36.46%, and the corresponding values of the LSC, SSC and IR regions reaching 34.28%, 29.99% and 42.48%, respectively.

We also constructed the phylogenetic trees with Neighbour-Joining (NJ, Phylip version 3.696, Felsenstein 2005), the maximum likelihood (ML, RaxML version 8, Stamatakis 2014) and Bayesian analysis (BI, MrBayes version 3, Ronquist and Huelsenbeck 2003) methods based on the alignments created by the MAFFT (Katoh and Standley 2013) using 9 related species of Fagales and the *Prunus persica* as outgroup (Figure 1). The results indicated that all three species from the Betulaceae were clustered together and *C. hebestroma* was most closely related to *O. rehderiana*. This complete chloroplast genome can be readily used for population genomic studies of *C. hebestroma*, and such information would be fundamental to formulate potential new conservation and management strategies for this endangered species.

**Figure 1.**
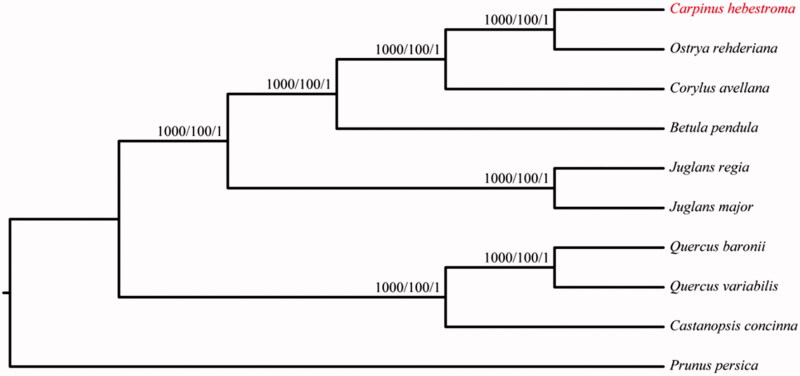
The phylogenetic tree based on the 10 complete chloroplast genome sequences. NJ/ML/Bayesian posterior probabilities/bootstrap values are shown at nodes. Accession numbers: *Betula pendula* LT855378, *Carpinus hebestroma* MG720819, *Castanopsis concinna* NC_033409, *Corylus avellana* KX822768, *Juglans major* NC_035966, *Juglans regia* NC_028617, *Ostrya rehderiana* KT454094, *Prunus persica* HQ336405, *Quercus baronii* KT963087 and *Quercus variabilis* KU240009.
